# Molecular Testing for Thyroid Nodules: The Experience at McGill University Teaching Hospitals in Canada

**DOI:** 10.3390/cancers14174140

**Published:** 2022-08-26

**Authors:** Mohannad Rajab, Richard J. Payne, Véronique-Isabelle Forest, Marc Pusztaszeri

**Affiliations:** 1 Department of Otolaryngology—Head and Neck Surgery, Jewish General Hospital, McGill University, 3755 Côte-Sainte-Catherine Road, Montreal, QC H3T 1E2, Canada; 2 Departments of Otolaryngology—Head and Neck Surgery, Royal Victoria Hospital, McGill University, 1001 Decarie Blvd, Montreal, QC H4A 3J1, Canada; 3 Department of Otolaryngology—Head and Neck Surgery, King Faisal Specialist Hospital & Research Center, Al Madinah Al Munawwarah 42523, Saudi Arabia; 4 Department of Pathology, Jewish General Hospital, McGill University, 3755 Côte-Sainte-Catherine Road, Montreal, QC H3T 1E2, Canada

**Keywords:** thyroid nodule, molecular testing, ThyroSeq, fine needle aspiration, Afirma, mutation, thyroid neoplasm, thyroid cancer

## Abstract

**Simple Summary:**

The aim of this review is to provide a general overview about the molecular markers (mutations and alterations) of thyroid cancers, present several molecular tests, and discuss the clinical applications of identifying these markers supported by the clinical experience of several high-volume thyroid cancer specialists at the McGill university teaching hospitals in Montreal, Canada.

**Abstract:**

In the past few decades, molecular characterization of thyroid cancer has made significant progress and is able to identify thyroid-cancer-related molecular markers that can then be applied clinically for improved decision making. The aim of this review is to provide a general overview about the molecular markers (mutations and alterations) of thyroid cancers, present several molecular tests, and discuss the clinical applications of identifying these markers supported by the clinical experience of several high-volume thyroid cancer specialists at the McGill university hospitals in Montreal, Canada. Our group experience showed that molecular testing can reclassify more than half of the patients with indeterminate thyroid nodules (Bethesda III and IV) into benign and spare these patients from unnecessary diagnostic surgery. Furthermore, it can help optimize the initial management in thyroid cancers with no evidence of high risk of recurrence of disease preoperatively. While routine molecular testing is not firmly established for thyroid FNA specimens that are suspicious or positive for malignancy (Bethesda V and VI), knowledge of a thyroid nodule’s molecular risk group profile in such cases, together with its clinical and radiologic features, can help select the optimal surgical options (lobectomy versus upfront total thyroidectomy and central neck dissection), as demonstrated by our studies.

## 1. Introduction

Thyroid nodules can be palpated in up to 5% of the population. On other hand, they are incidentally detected by high-resolution ultrasound (US) in 19–68% of random individuals. The likelihood of malignancy in these nodules can be as high as 10–15% [1]. Determining whether a thyroid nodule is benign or malignant is at times a diagnostic challenge. Indeed, thyroidectomy (partial or total) has been the gold standard to obtain histopathological confirmation of malignancy. Nevertheless, up to 90% of patients undergoing diagnostic surgery end up with benign disease and therefore will have undergone unnecessary surgery. While thyroid surgery is usually well-tolerated, there are significant risks and morbidities involved with this procedure such as: risk of surgical complications (recurrent laryngeal nerve injury, hematoma, hypocalcemia, scar, etc.), reduced quality of life, and possibly lifelong hormonal supplementation or replacement [2,3]. There is also a financial cost to the patient and the healthcare system as a whole for the unnecessary surgery, which have economic consequences, specifically but not exclusively in countries with publicly funded healthcare systems.

In the past few decades, the evaluation and management of thyroid nodules has significantly evolved. In 1967, the ultrasound (US) was introduced as a diagnostic tool for thyroid nodules. This helped to identify certain features associated with higher probability of thyroid cancer [4]. Fine needle aspiration (FNA) cytology became widely available in the 1970s in Asian countries and in the 1980s in the United States [5]. Nowadays, US and cytological analysis are considered common practice for the evaluation of thyroid nodules [1]. US is useful in detecting thyroid nodules with suspicious features that require further evaluation by FNA. Cytological evaluation is used to provide risk stratification by successfully classifying 70–80% of the thyroid nodules as benign or malignant [6]. Multiple reporting systems have been proposed for both US and FNA [1,7,8,9,10,11]. The most common systems are the Thyroid Imaging Reporting & Data System for the US and The Bethesda System for Reporting Thyroid Cytopathology for the FNA [10,11]. Even though the use of FNA has successfully decreased the number of thyroid nodules undergoing diagnostic surgeries, 20–30% of thyroid nodules are classified as indeterminate with a risk of malignancy ranging from 15% to 65% [12].

In the past decade, molecular characterization of thyroid cancer has made significant progress. This tool allows for the identification of thyroid-cancer-related molecular markers which can then be applied clinically for improved decision making [13]. The most widely accepted theory for thyroid carcinogenesis is through the transformation of follicular cells into differentiated or undifferentiated thyroid cancer which occurs through a series of steps that involve various genetic alterations. Early driver alterations are usually responsible for initiating the cancer process and secondary or promotor alterations are driving progression from differentiated to undifferentiated cancer [14,15]. These molecular alterations are classified into different groups, each group is associated with different gene expression and signaling pathways, leading to different clinical characteristics, histophenotypes and prognosis [16,17]. This concept has been successfully applied by different molecular tests to provide useful diagnostic, prognostic, and therapeutic information that can impact on the management and decision-making of patients with thyroid nodules and cancers.

The aim of this review is to provide a general overview about the molecular markers (mutations and alterations) of thyroid cancers, present several molecular tests, and discuss the clinical applications of identifying these markers supported by the clinical experience of several high-volume thyroid cancer specialists at McGill university hospitals in Montreal, Canada.

## 2. Molecular Markers

According to the 2022 World Health Organization (WHO) classification, the follicular cell derived malignant neoplasms include: papillary thyroid carcinoma (PTC), follicular thyroid carcinoma (FTC), invasive encapsulated follicular variant of papillary thyroid carcinoma (IEFVPTC), oncocytic thyroid carcinoma (OTC), high-grade follicular-derived carcinoma (poorly differentiated thyroid carcinoma (PDTC) and differentiated high-grade thyroid carcinoma), and anaplastic thyroid carcinoma (ATC). The newly described entity, non-invasive follicular thyroid neoplasm with papillary like nuclear features (NIFTP), was classified as low-risk neoplasm along with thyroid neoplasms of uncertain malignant potential, and hyalinizing trabecular tumors. The only thyroid C-cell- derived neoplasm is medullary thyroid carcinoma [18].

**Figure 1:**
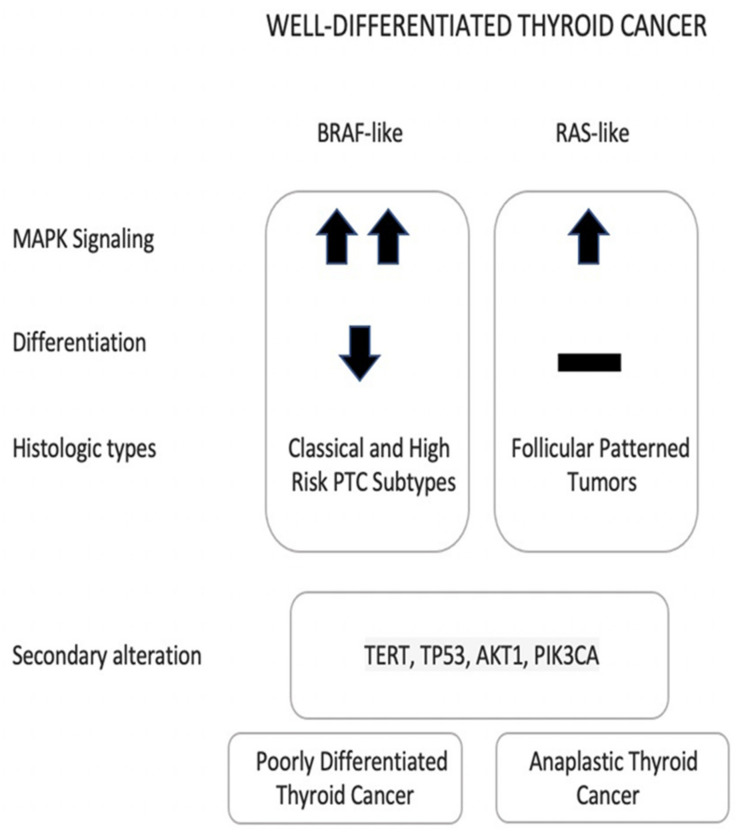
The Consequences of BRAF-like and RAS-like Molecular Alterations in Thyroid Carcinoma. Abbreviations: (↑): Upregulated MAPK pathway output in RAS-like tumors; (↑↑): BRAF-like tumor has a Higher MAPK pathway output than RAS-like tumors; (↓): Less differentiated; (-): Retained differentiation. Acquiring secondary alterations results in dedifferentiation and aggressive behavior.

The molecular pathogenesis of all thyroid carcinomas has been significantly explored in recent decades [19,20,21,22,23,24,25,26,27]. Most of thyroid cancer driver alterations cause dysregulation of the mitogen-activated protein kinase (MAPK) and phosphatidylinositol-3 kinase (PI3K)-AKT pathways. Both pathways include multiple effectors that are responsible for transmitting signals from the cell receptor to the DNA, to regulate various cellular processes, such as cell proliferation, growth, and apoptosis [28,29]. Novel genetic alterations not related to this pathway have been discovered in the Thyroid Cancer Genomic Atlas (TCGA) cohort. However, thyroid cancer is still considered a stable malignancy with low-frequency molecular alterations in comparison to other carcinomas [22]. The molecular signature among and within different thyroid cancers are variable and may affect the patients’ prognosis. The existence of certain secondary molecular alterations (e.g., *TP53*, *TERT*) is usually associated with an increased risk of having a more aggressive and dedifferentiated type of thyroid cancer [15,30] (Figure 1).

### 2.1. Papillary and Follicular Thyroid Cancers

The genotype-phenotype correlation between two groups of mutations and the morphologic features of differentiated thyroid cancers has been explored in multiple molecular studies [18,20,31,32,33,34,35,36,37] (Figure 1). Both groups are activating the MAPK pathway but do so to different degrees. RAS-like mutations result in lower MAPK pathway signaling due to their response to the negative feedback. They are associated with more differentiated tumors that are usually encapsulated, rarely spread to lymph nodes (LN), retain their expression of iodine-metabolism genes, and are associated with an expansile growth pattern with a follicular architecture, with or without nuclear features of PTC. On the other hand, BRAF-like mutations result in higher MAPK pathway signaling due to their unresponsiveness to the negative feedback. They are rarely encapsulated tumors and have a higher risk of spread to LN, suppressed iodine-metabolism genes, and infiltrative growth pattern with well-developed nuclear features of PTC. It is also worth mentioning that the PI3K/AKT pathway is activated in RAS-like tumors while it is not activated in BRAF-like tumors [15,18,22].

According to the WHO classification of endocrine tumors, PTCs are defined as malignant tumors deriving from follicular cells that present specific nuclear features with either papillary or solid/trabecular architecture, or evidence of invasion in follicular patterned tumors [18]. Generally, they are associated with >90% 10-year survival, however, the prognosis relies on the associated molecular and clinicopathological features [38]. PTCs can present as different subtypes with classical PTC (C-PTC) being the most common subtype [17]. Aggressive subtypes like tall cell (TC-PTC), columnar cell (CL-PTC) and hobnail (H-PTC) PTCs, are associated with higher risk of invasive histological findings, hence they are associated with worse prognosis and higher risk of recurrence [39,40]. Solid (S-PTC) and diffuse sclerosing (DS-PTC) subtypes were highlighted in the WHO 5th edition of thyroid neoplasm and they can be considered aggressive tumors [18,41,42]. *BRAF-V600E* is the most common mutation in PTC (45–50%), it can be found in 70% of C-PTC and 90% of TC-PTC. Gene rearrangements, including *RET* and *NTRK1/3* fusions (10% and 5%), are well known driver alterations for PTCs and are usually found in DS-PTC and S-PTC, especially in radiation induced cases. *ALK* fusions (1%) are another rare BRAF-like molecular alterations that are mainly associated with aggressive PTCs subtypes including DS-PTC (13%). All these molecular alterations can be found interchangeably in different aggressive PTCs subtypes, while they are very rarely found in the follicular patterned tumors (almost exclusively in the infiltrative follicular variant of PTC (IFVPTC)). *TERT* promoter mutation and *TP53* can be present in PTCs (10% and 3.5%) and can be found with a higher frequency in morphologically aggressive tumors. Several novel molecular alterations were identified in the TGCA study including: *EIF1AX* mutation, DNA repair genes (*PPM1D, CHEK2, S2D* and *S2E*), and tumor suppressor genes (*RB1, NF1, MEN1* and *PTEN*) [15,18,39].

The follicular variant of PTC (FVPTC) is the second most common PTC subtype after C-PTC [17]. It has two distinct variants with different growing patterns and behaviors: the IFVPTC resemble PTC with infiltrative growth pattern and propensity to spread to the LNs, while the infiltrative encapsulated FVPTC (IEFVPTC) resembles FTC with expansile growth patterns and propensity for distant metastasis. Molecular studies have shown that IFVPTC is a BRAF-like neoplasm, while the IEFVPTC is a RAS-like neoplasm [18]. Due to its indolent behavior, the noninvasive encapsulated FVPTC was reclassified as NIFTP which is considered as a very low-risk neoplasm or as a premalignant lesion that does not need any further treatment beyond hemithyroidectomy which is required for its definitive diagnosis [43]. Both NIFTP and IEFVPTC are RAS-like neoplasms harboring *RAS* mutation in 40–70% of cases and cannot be differentiated from each other on molecular basis [15,18,38,44]. *THADA::IGF2BP3* fusion and *PAX8::PPARG* rearrangements were found to be associated with these follicular patterned tumors as well [27,45].

FTC is an invasive follicular cell derived malignant tumor that lacks PTC nuclear features [18]. It accounts for 6% to 10% of all thyroid cancers. Several studies explored the molecular signatures of FTC, and they have shown genetic similarities between IEFVPTC, NIFTP, and FTC. In addition to the well-known high prevalence of *RAS* and RAS-like mutations in FTC, *PAX8::PPARG* is seen in up to 30–40% of cases. *PTEN, DICER1* and *EIFA1X* can also be found FTC [26,46]. *TERT* promotor mutation was reported to be found in 15% of FTCs [14].

### 2.2. Oncocytic Carcinoma of the Thyroid (OCT)

OCTs are defined as invasive follicular derived malignancy that consist of at least 75% of oncocytic cells without PTCs nuclear and high-grade features [18]. They account for 3-5% of all thyroid cancers. OCTs used to be classified as a subtype of FTC. However, since the discovery of their completely different molecular profile, OCTs are now considered an independent entity, distinct from FTCs. The molecular signature of OCTs is characterized by three main alterations: mitochondrial DNA alterations in complex 1, somatic nuclear DNA mutations including: *DAXX, EIFA1X, NF1, TP53, NRAS* and *TERT* promoter, and chromosomal alterations including haploid phenotype, polysomy and/or duplication of chromosomes 7, 5, and 12. Mutation in *TERT* promoter and *DAXX*, and near haploid state (except for chromosome 5 and 7) with loss of heterozygosity are more likely to be found in OCTs with aggressive behavior [15,19,20].

### 2.3. Advanced Differentiated Thyroid Cancer, PDTC and ATC

Compared to non-advanced well-differentiated thyroid cancers (PTC, FTC and OCT), advanced differentiated thyroid cancer, PDTC and ATC usually harbor multiple molecular alterations (Figure 1). While *BRAF* and *RAS* mutations are still common alterations in PDTC and ATC, these tumors are mainly associated with increased frequency of *TP53* mutation, which can be found in 20–30% of PDTC and 28–73% of ATC, *TERT* promoter mutation, which can be found in 33–44% of PDTC and 43–73% of ATC, and *PIK3CA* mutations. *EIF1AX* is found with higher frequency in PDTC and ATC (9–14%), when compared to the PTCs, where it is found only in 1% of all the cases. Alterations in cell cycle regulation genes (CDKN2A, CDKN2B, and CCNE1), histone regulation genes, and SW1/SNF complex can also lead to ATC [14,15,17,47,48,49]. The acquisition of secondary mutations is believed to lead to dedifferentiation and aggressive behavior [15,44]. PDTC can be defined using two criteria: Turin or Memorial Sloan Kettering Cancer Center (MSKCC) criteria. The frequency and the type of the molecular alterations can vary according to the used criteria. BRAF-like mutations are more frequent in tumors that are diagnosed using MSKCC, whereas RAS-like mutations are more common in tumors that are diagnosed with the Turin criteria, [14,47]. ATC is a highly aggressive tumor with variable undifferentiated cellular components and a high mutation burden that often arise in a background of differentiated thyroid cancer [18].

There is no consensus on the definition of advanced differentiated thyroid cancer, however, persistent/recurrent disease, disease resistant to radioactive iodine (RAI), advanced stage, bulky disease, high-grade and distant metastasis are considered hallmarks of advanced disease [18,50]. The presence of secondary mutations is also more common in advanced PTCs when compared to non-advanced PTCs (7% vs. 2.5%). In addition, advanced PTCs are more likely to harbor mutations like *TP53*, *PIK3CA,* and *TERT* promoter mutation [15,19,20].

## 3. Molecular Tests

Advances in thyroid tumor molecular characterization have resulted in the development of multiple molecular commercial tests to supplement cytology and US and improve risk stratification and management decision-making for thyroid nodules [51] (Table 1). The molecular tests are categorized as either a rule in or rule out tests depending on their capacity to either confirm or exclude malignancy [51]. To rule out malignancy, a test needs to have a high sensitivity and a high negative predictive value (NPV). On the other hand, high specificity and high positive predictive value (PPV) are required to rule in malignancy. In a population with prevalence of cancer among indeterminate thyroid nodules between 20% to 40%, it was estimated that a NPV of 94% and a sensitivity of 90% are required to rule out malignancy, while a specificity of 80% and PPV of 60% are needed to rule in malignancy [52]. Hence, a rule in test will perform better in a context where the prevalence of malignancy is high, helping to refine the surgical management, to identify more aggressive cancers and to predict the prognosis and outcome. A rule out test will perform better when the risk of malignancy is low and will help to avoid diagnostic surgeries on benign thyroid nodules. Nowadays, multiple tests can identify specific thyroid-related molecular alterations, which can contribute further to more individulaized decision making and management for thyroid nodules and cancers [53,54,55]. In general, genotyping-based tests for thyroid FNAs do not provide a binary “negative” or “positive” result. Instead, such tests offer a gradient of cancer probability (and information suggesting tumor type and prognosis as well) based on the type, number, and allelic frequency of the molecular alteration(s) that are identified (See Supplementary File S1). The well-characterized associations between a tumor’s molecular profile and its histophenotype and prognosis permit stratification of tumors into low-, intermediate- and high- molecular risk groups (MRG). Typically, the low-risk MRG is represented by a single *RAS* mutation or *RAS-like* variant. The intermediate-risk MRG includes the *BRAF V600E* mutation, other BRAF-like variants, or copy number alterations. The high-risk MRG profile is characterized by the co-occurrence of one of the aforementioned driver alterations with mutations in genes such as *TERT, TP53, AKT1,* and/or *PIK3CA*; this profile helps identify a sub-group of thyroid cancers with unfavorable outcomes. Some of the commonly used commercially available molecular tests are ThyroSeq genomic classifier, Afirma gene sequencing classifier and Xpression Atlas (GSC/XA), and ThyGenNEXT and ThyraMIR.

### 3.1. ThyroSeq v3

The third version of ThyroSeq molecular test (ThyroSeq v3) is the most updated version of the test. It utilizes the next generation sequencing technique to evaluate 112 thyroid-related genes for wide spectrum molecular alterations which includes point mutations, gene fusions, copy number alterations (CNAs) and gene expression alterations (GEAs). Unique genomic classifier is used to analyze the results and reports it as negative or positive. The risk of malignancy and cancer recurrence are estimated in the positive nodules according to the detected molecular alterations [59]. According to Steward et al. validation study, the performance of the test is 94% sensitivity, 82% specificity, 97% NPV and 66% PPV with a 61% benign call rate (BCR) [53].

### 3.2. Afirma GSC/XA

Afirma GSC is the updated version of the Afirma gene expression classifier (GEC) which was designed as a rule out test. To improve the PPV and the specificity of the test, the GSC was developed. It is based on the RNA whole-transcriptome sequencing technology to detect previously undetectable molecular information which include components for *BRAF V600E* mutation, *RET* fusion, parathyroid tissue and MTC. It utilizes machine learning algorithm to analyze the results and report it as benign or suspicious [2]. In the validation study, it was demonstrated that the GSC has 91% sensitivity, 68% specificity, 96% NPV and 47% PPV with a 54% BCR [56]. Using the same technology, the Afirma XA can detect expressed variants and fusions in the nodules with suspicious GSC result or Bethesda V/VI. Furthermore, it provides more detailed information about the risk of malignancy, neoplasm type, variant class, and available systemic therapy which might help for more personalized management and decision making in those patients. However, Afirma XA is unable to identify *TERT* promoter mutation, which is located in the promoter region, henceforth, it is not amendable for detection using RNA sequencing technology [54,57].

### 3.3. ThyGenNEXT/ThyraMIR

ThyGenNEXT/ThyraMIR uses targeted sequencing for five gene mutations and three gene fusion transcripts with a unique 10-miRNA GEC that categorizes thyroid nodules according to risk of malignancy to either high or low risk nodules based on microRNA profile. When ThyGenNEXT is negative for genetic changes or a mutation with a reduced specificity for malignancy is discovered, ThyraMIR testing is automatically activated. 197 Bethesda III/IV thyroid nodules were included in the clinical multicenter blinded validation trial. With a population prevalence of malignancy of 30%, the sensitivity was 95%, the specificity was 90%, the NPV was 97%, and the PPV was 75% [15,55].

## 4. Clinical Utility and Our Group’s Experience

**Figure 2:**
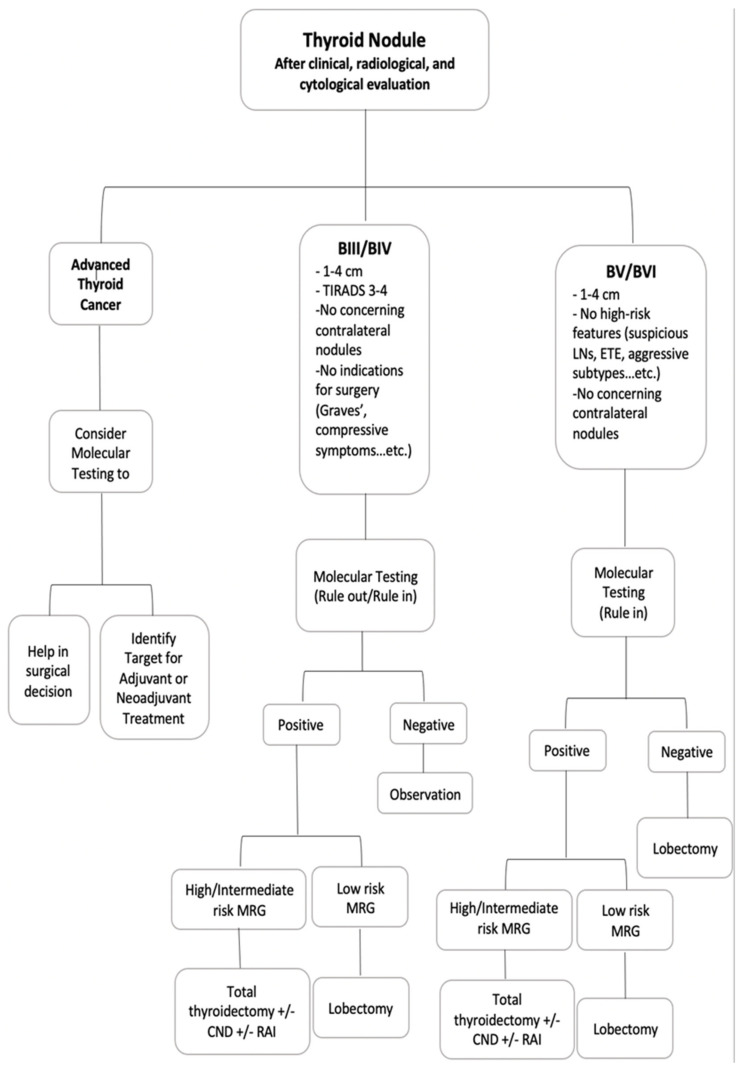
Our group algorithm for using molecular testing in thyroid nodules. Abbreviations: BIII: Bethesda III; BIV: Bethesda IV; BV: Bethesda V; BVI: Bethesda VI; MRG: molecular risk groups; CND: Central neck dissection; RAI: Radioactive Iodine Treatment.

The performance of molecular tests is usually determined by using different measures which include analytical validity, clinical validity, and clinical utility [1]. The analytical validity is the ability of the test to accurately identify the molecular alterations in different technical settings. The clinical validity is the ability of the test to distinguish different groups of patients by either ruling them in or out [60]. These two measures are very important for the test to perform well, however, the most important factor for the test to be included in the routine clinical practice is its ability to positively affect the clinical outcome and management decision making (clinical utility) [61]. The molecular tests can be used in different clinical settings (Table 1). It can be used for Bethesda III and IV nodules to identify nodules with low risk of malignancy and avoid diagnostic lobectomies on benign thyroid nodules. It can be used in biopsy proven cancers to assist in the decision of the extent of the surgery (lobectomy, total thyroidectomy, central neck dissection, etc), and also, it is used in advanced thyroid cancers to identify targetable molecular alterations [1,11,50,62]. While the analytical and clinical validity of different molecular tests have been well established through multiple studies, data regarding their clinical utility is still lacking [51,53,55,56,59,61]. Here, we present the clinical applications of identifying the molecular markers in thyroid nodules supported by the clinical experience of several thyroid specialists at McGill university teaching hospitals (Figure 2).

### 4.1. Bethesda III and IV Nodules (Indeterminate Thyroid Nodules)

The current aim of using molecular tests for indeterminate thyroid nodules (ITNs) is to avoid unnecessary surgery when managing patients with Bethesda III/IV results. Instead of diagnostic surgery in this patient population, where the majority of nodules are benign, a negative molecular test allows for safe clinical follow up in the majority of cases. Multiple guidelines suggest the use of molecular testing as an adjunct to clinical, radiological and cytological findings to refine the risk of malignancy [1,9,11,62]. According to The National Comprehensive Cancer Network (NCCN) Guidelines, if molecular testing indicates a risk of malignancy equivalent to the risk that is seen in benign thyroid nodule FNA (about 5% or less), clinical follow-up of the thyroid nodules can be considered [63]. To achieve this goal, a test with high sensitivity and NPV should be used. In order to determine which patients with Bethesda III/IV thyroid nodules will benefit most from molecular testing at our center from a cost analysis perspective, specific guidelines have been introduced. Each of the following criteria must be met in the guidelines: thyroid nodules measuring 1 to 4 cm, TIRADS 3 and 4 on US, either two Bethesda III results or one Bethesda IV result on cytology, and the patient will choose to undergo surveillance if the molecular test result is negative. After receiving the cytological diagnosis in patients who meet the criteria, management options including the molecular testing are discussed with the patients [1]. The rationale of doing the molecular testing in these patients is to avoid unnecessary surgeries, to improve the risk of cancer stratification and to perform the optimal extent of surgery if the test is positive.

In our experience, using molecular testing decreases the need for diagnostic surgery in the patients with ITNs [64,65]. In Kay-Rivest et al. study, 172 patients with ITNs who underwent Afirma GEC were investigated. In total, 89 nodules (52%) were benign as per Afirma GEC. Surgery was performed for the remaining patients with suspicious results (48%) and half of the nodules were malignant. In this study, a suspicious result with Afirma GEC increased the risk of malignancy in ITNs to 50% [64]. In Chen et al., among the 50 nodules with Bethesda III/IV results who underwent ThyroSeq v3, 20 nodules were positive, 24 nodules were negative, 4 nodules were currently negative, and 2 nodules were negative but limited. They obtained a 58% BCR, depending on considering both negative and currently negative nodules as benign result [65]. Of note, both studies obtained benign results through molecular testing in more than half of the patients with ITNs. Conventionally, most of these patients would have had diagnostic surgery. Instead of surgery, these individuals were handled with frequent follow-up. Nonetheless, long-term follow up data is not available.

ThyroSeq v3 provides further details about the involved molecular alterations that can be useful in the management decision making and might affect the extent of the surgery [53]. Interestingly, all the 20 ITNs with positive ThyroSeq v3 results in Chen et al. were low-risk cancers that had RAS-like mutations (*NRAS, HRAS, PTEN, EIF1AX,* and *THADA::IGF2BP3* fusion), CNAs or GEAs, with *RAS* mutation (*NRAS* & *HRAS*) being the most common (60%) [65]. Similar findings were found in the validation study for ThyroSeq v3, most of the ITNs (84%) in their study were low-risk cancers with RAS-like mutation (57%), CNAs (21%), and GEAs (8%). However, 14% were associated with BRAF-like mutations (12%) (*BRAF V600E*, *NTRK3* fusion, *RET* fusions and *BRAF* fusions) or high-risk mutations (2%) (*TERT* promotor mutation & *TP53* mutation) [53]. The data from the validation studies of the different molecular tests are showing that most of the ITNs after surgical resection are either benign, NIFTP or well-differentiated thyroid cancer with excellent prognoses [66]. There is a risk of having aggressive cancer in ITNs which is usually associated with the presence of aggressive mutations like BRAF-like mutations or high-risk mutations (e.g., *TERT* or *TP53*) [67]. Interestingly, a study by Turkodgan et al. showed that in surgically resected ITNs, Bethesda III nodules are more likely to be aggressive than Bethesda IV nodules and molecular testing is strongly correlated with a higher risk of malignancy and aggressive features in these nodules [68]. This indicates that preoperative molecular testing is not only valuable to avoid unnecessary surgery, but also to help guiding the extent of the surgery in the ITNs with positive results, since some mutations are associated with more aggressive cancers.

*RAS* and RAS-like mutations are not specific to malignant tumors. *RAS* mutation has been reported to be detectable at all stages of thyroid tumor development. According to some studies, it can be found in 33% of follicular adenomas, 53% of FTCs, and 60% of ATCs [69]. In follicular cell originated tumors, RAS-like mutations are associated with low MAPK pathway signaling that essentially lead to development of benign or low-risk thyroid tumors like FA, NIFTP, FVPTC and FTC, which cannot be reliably distinguished on cytology alone and are usually reported as an ITNs (Bethesda III or IV) [17,45,70]. Two studies that were conducted by our group found that there is a significant association between thyroid nodules with Bethesda III and IV results and RAS-like mutations (including *EIFA1X* and *PTEN* mutations) [71,72]. According to multiple recent studies, when isolated RAS-like mutations are detected in thyroid nodules, they are associated with low-risk tumors and favorable prognosis [45,70,73,74,75]. However, supported by the finding that these mutations can be found in all stages of thyroid tumor development, it is hypothesized that these mutations can drive the progression from follicular adenoma to carcinoma to further dedifferentiated cancer when associated with other molecular alterations. Based on this evidence, it is justifiable to surgically remove these nodules that harbor these mutations to prevent further progression even if they are found to be benign on final postoperative histopathological examination [76].

### 4.2. Bethesda V and VI Nodules

Multiple studies have been done by our group to explore the risk of aggressive features with certain mutations in histopathological proven cancers. In all these studies, the tumors were considered to be aggressive if one or more of the following features were present: macroscopic extrathyroidal extension (ETE), lymph node metastasis (LNM), extranodal extension (ENE), PDTC, and high-risk histological features (TC-PTC, CL-PTC, H-PTC, S-PTC, DS-PTC). The impact of these features on the prognosis of the patients with thyroid cancer has been clearly demonstrated by multiple studies, which showed that the presence of these features is associated with worse overall survival (OS), disease specific survival (DSS) and increased risk of recurrence [40,42,77,78,79,80]. To help management and treatment, the ATA guidelines classify all the tumors with these features in the intermediate and high-risk for recurrence groups, and more extensive treatment (including total thyroidectomy and RAI treatment) was recommended [1]. Most of these features are difficult if not impossible to detect in the preoperative setting, as a result, this issue leads to suboptimal initial treatment at times with an initial thyroid lobectomy followed by a completion thyroidectomy as a second operation [81]. Molecular testing can be a useful tool for predicting these features preoperatively and can assist in uncovering the phenotype of the thyroid nodules and cancers and thus perform the optimal extent of surgery at the initial setting.

Unlike ITNs, the main clinical benefit of molecular testing in these nodules (Bethesda V and VI nodules) is not to predict the risk of malignancy but to preoperatively identify those with higher risk to be aggressive and treat them optimally, in one surgical setting. In a study from Krasner et al., 103 cases who had ThyGenNEXT molecular testing and surgery were analyzed. The patients were divided into three groups, Group 1 (*BRAF V600E, RET::PTC1* and *TERT*) was associated with higher risk of aggressive features (65.7%) when compared to Group 2 (*NRAS, HRAS, KRAS, BRAF K601E* and *PAX8::PPARy*) and Group 3 (no mutation) which were associated with 21.7% and 11.1% risk of aggressive features, respectively [82]. Another study found that *BRAF V600E*, *RET* fusion and *TERT* promoter mutation are associated with higher risk of aggressive features when compared to the other molecular alterations (*RAS-type*, *EIF1AX*, CNAs, and GEAs) [71]. The result of both studies is supporting that BRAF-like mutations and *TERT* promoter mutation are associated with higher risk of aggressive features than RAS-like mutations, CNAs and GEAs and can benefit from more extensive surgical management.

A *BRAF V600E* mutation is the most common mutation in thyroid cancer (35–70% of PTCs). It has been shown to be closely associated with TC-PTC and C-PTC and to correlate with aggressive tumor features including ETE, LNM, ENE and distant metastasis [71]. A recent metanalysis showed that *BRAF V600E* mutation is associated with higher likelihood of recurrence even in papillary thyroid microcarcinoma (PTMC) [83]. However, the ATA guideline from 2015 considers PTMC with *BRAF V600E* mutation to be associated with low risk of recurrence which may lead to suboptimal treatment [1]. Two studies were conducted to analyze the relation of the nodule size to the *BRAF V600E* mutation. In both studies, the presence of a *BRAF V600E* mutation in the nodules was associated with aggressive features regardless of the size of the nodule [36,37].

Finally, a multicenter study investigated the role of molecular testing in optimizing the extent of surgery in patients with Bethesda V and VI thyroid nodules with no signs of high-risk features before the surgery. The main outcome was to identify how many patients had the optimal surgery done when using molecular testing compared to patients without molecular testing (for example: if a lobectomy was the surgery planned and performed, no completion was necessary following final histopathological analysis). It was found that 91.86% of the patients in the molecular group had an optimal surgery while it was only 61.11% in the group without molecular testing [58]

### 4.3. Advanced Thyroid Cancers

**Figure 3:**
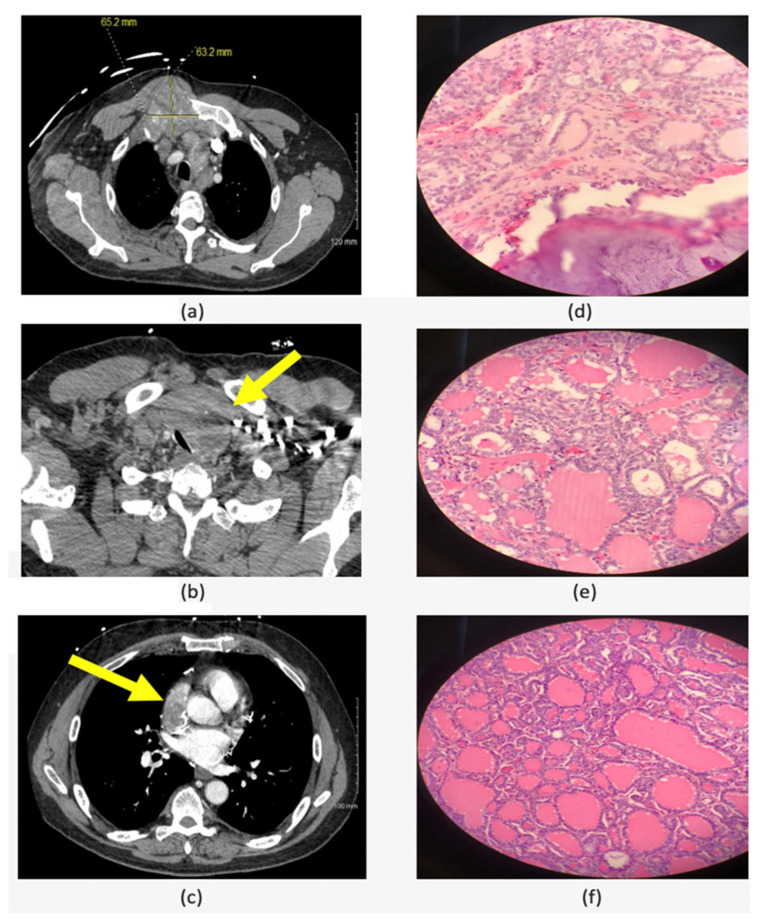
Left column is showing the neck and chest CT scan: (**a**) 6.5 cm right manubrial costal junction mass; (**b**) left thyroid lobe mass with tracheal compression (**c**) 5.1 cm superior vena cava (SVC)/right arterial mass. Right column is showing the final pathology from all the resected sites which showed follicular variant of papillary thyroid carcinoma: (**d**) chest wall metastasis; (**e**) left thyroid tumor; (**f**) right atrium metastasis.

The criteria to define advanced thyroid cancer and the indications for systemic targeted treatment have been discussed in details in the American Head and Neck Society Endocrine Surgery Section and International Thyroid Oncology Group consensus statement on mutational testing in thyroid cancer: Defining advanced thyroid cancer and its targeted treatment [50]. The main goal for molecular testing in advanced thyroid cancer and anaplastic thyroid cancer is to detect targetable alterations and identify the optimal treatment for these patients [84]. After doing the molecular testing, multikinase inhibitors or systemic treatment targeting certain alterations (e.g., immune checkpoint inhibitors) can be given to the patients with progressing or symptomatic advanced differentiated thyroid cancer who had failed traditional therapy as neoadjuvant treatment in the presence of pre-operative advanced local disease [50] and as adjuvant therapy for selected patients with ATC [84]. One of the advanced thyroid cancer cases that was treated in our center is a 53-year-old male patient who presented with chest pain and chest wall mass. CT scan showed 6.5 cm right manubrial costal junction mass, 5.1 cm superior vena cava (SVC)/right arterial mass and left thyroid lobe mass (Figure 3). The biopsy of the chest wall was consistent with metastatic well-differentiated thyroid carcinoma and the thyroid USFNA showed PTC. Molecular testing (ThyroSeq v3) was positive for *NRAS* and *TERT* promoter mutations. According to the literature, the prognosis of cardiac metastasis from thyroid carcinoma is extremely poor [85]. However, since tumors with combined *NRAS* and *TERT* promoter mutations are not expected to cause local tissue invasion and are, therefore, potentially resectable, it was decided to proceed with surgical resection, followed by external beam radiation and RAI treatment. Final pathology from all the resected sites (thyroid, right atrium, SVC and chest wall tumors) was positive for FVPTC (Figure 3). Currently (23 months after surgery), the patient is doing well and had a partial response to the radiotherapy and three 150 mCi doses of RAI treatment with minimal progression of the bone metastasis. This case exemplifies that identifying molecular markers in advanced thyroid cancer can also play a role in the surgical decision in addition to detecting a possible targetable alteration/mutation.

## 5. Discussion

In recent decades, molecular characterization has been extensively studied [15,24]. Various molecular tests and genomic classifiers have been developed to provide more useful information to manage ITNs and thyroid cancers [53,55,56]. Furthermore, studies have correlated certain mutations with different cancer types and characteristics [22,71,82]. The BCR is defined as the percentage of the ITNs that were classified as benign [86]. It is important to understand that the performance of molecular tests, as a rule out or a rule in test (NPV and PPV), is affected by the prevalence of thyroid cancers among the ITNs of the population it is used for. Indeed, multiple validation studies demonstrated the ability of different types of molecular testing in achieving BCR that is ranging between 41% to 61% in an environment with a prevalence of 20–40% thyroid cancers among ITNs [53,56,87]. As a result, the ATA urges thyroid specialists to discover the prevalence of thyroid cancer in each Bethesda category in their tested patient population and to assess how the local thyroid cancer prevalence may influence the accuracy of molecular testing (PPV and NPV) when used in their specific clinical practice [88].

Two studies that were done in our centers showed that Afirma GEC and ThyroSeq v3 were able to obtain 52% and 58% BCR, respectively, managing to spare more than half of the patients from unnecessary diagnostic surgeries [64,65]. The 2015 ATA guidelines recommended the following criteria to consider lobectomy in the patients with 1–4 cm differentiated thyroid cancers which include: no ETE, no suspicious LNs and the absence of family history of thyroid cancer or radiation exposure [1]. Multiple studies showed that despite the strict preoperative selection and adherence to the ATA guidelines, 35–65% of individuals with a low-risk 1–4 cm PTC who originally met the requirements for a lobectomy would eventually need to have their entire thyroid removed [89,90,91,92]. One of the studies that was done by our group showed that the thyroid cancers with *BRAF V600E, RET::PTC1 and TERT* promoter mutations were associated with higher risk of aggressive features (65.7%) when compared to the thyroid cancers with *NRAS, HRAS, KRAS, BRAF K601E* and *PAX8::PPARG* molecular alterations and thyroid cancers with no mutation, which were associated with 21.7% and 11.1% risk of aggressive features, respectively [82]. Subsequently, another study was done to investigate if using the molecular testing preoperatively in the patients with Bethesda V and Bethesda VI thyroid nodules with no evidence of high-risk disease can optimize the extent of surgery. The study showed that 91.86% of the surgeries in the molecular group was optimal while it was only 61.11% in the group without the preoperative molecular testing [58]. This finding shows that using molecular testing in these patients decreases the chances of under or overtreatment from 38.89% to 8.14%. Another recent retrospective study from Pittsburgh by Skaugen et al. investigated the benefits of ThyroSeq v3 in 128 Bethesda V nodules, and their results also suggest that molecular testing may facilitate more individualized management of patients with Bethesda V nodules by guiding the extent of surgery and/or identifying possible non-surgical therapies [93]. There is an ongoing prospective pilot study from the same Institution that is being conducted to evaluate the ability of molecular testing to direct extent of initial thyroid surgery; this study is expected to end in February 2023. Molecular testing currently plays a significant role in improving understanding and management of ITNs, and with deeper understanding of the histopathological features associated with any given mutation or group of mutations, it may help tailor the extent of surgery and better guide treatment of thyroid cancers with no evidence of high-risk features preoperatively, in addition to several other potential benefits (Table 1).

Clinical practice for thyroid nodules and cancer varies greatly across countries in terms of disease prevalence, diagnostic procedures, diagnostic test availability, conservative therapeutic strategy, health insurance policy, and health care governmental legislation [5]. Some Asian countries like South Korea and Japan have their own reporting system for reporting US findings with their own FNA indications and recommendations [8,94]. TBSRTC is widely used for reporting thyroid cytology in Asia, except Japan, where the general rules for description of thyroid cancer (GRDTC) and the national reporting system for thyroid FNA cytology (Japanese system) are more widely used. The Japanese system is preferred in the centers with high-volume thyroid surgery because of its ability to furtherly stratify the risk of malignancy (ROM) in patients with follicular neoplasm into three subcategories to reduce unnecessary diagnostic surgeries [95]. Furthermore, unlike other countries where surgery is preferred, patients with ITNs in these countries are more prone to have active surveillance (AS) and after strict triage based on clinical, radiological, and cytological finding, the decision for surgery will be only offered to those with high ROM [96,97]. According to a meta-analysis by Vuong et al., in most cytological categories, with the exception of the malignant category, the ROM of resected nodules was much greater in Asian practice than in Western practice [97]. In such an environment, because the prevalence of cancer is high among the ITNs in Asian countries, the performance of the molecular testing as a rule out test will not be optimal. Hu et al. used a unique molecular platform comprised of RNA and DNA-RNA classifiers, comparable to Afirma GEC and ThyroSeq v3, to assess the role of molecular testing in Asia. The molecular testing was done for 140 patients with ITNs and 58 nodules were resected based on clinical features, radiological characteristics and patient willingness rather than the molecular testing. Among the 58 ITNs that had molecular testing and were removed surgically, 43 nodules were malignant or borderline tumors and 15 were benign in the final pathology with 74.1% ROM. For the RNA classifier, the sensitivity, specificity, PPV, and NPV were 93%, 40%, 81.6% and 66.7%, respectively. For the DNA-RNA classifier, the sensitivity, specificity, PPV, and NPV were 88.4%, 53.3%, 84.4% and 61.5%, respectively. This study showed that the PPV for the DNA-RNA and the RNA classifier were higher (84.4% and 81.6% vs. 37.4% and 63.3%) and the NPV were lower (61.5% and 66.7% vs. 94.3% and 90.9%) than those of Afirma GEC and ThyroSeq v3 in their cohort [98]. The result of this study is showing that in an environment with high ROM among ITNs, like these Asian countries, the molecular testing may be more useful as a rule in test rather than a rule out test.

The low-risk PTMC can be treated with different management options. AS is the preferred management option for those patients as recommended by the Japan Association of Endocrine Surgeon (JAES). Two studies that were done in Japan showed the safety of AS with low risk of tumor progression and LN metastasis and the safety for surgical conversion if indicated [99]. The JAES recently published their indications and strategy for AS for low-risk PTMC [100]. Minimally invasive treatment (MIT) is another option that can be used for those tumors as recommended by European Thyroid Association and Cardiovascular and Interventional Radiological Society of Europe 2021 Clinical Practice Guideline for the Use of Minimally Invasive Treatments in Malignant Thyroid Lesions. It is worth to note that they mention that the presence of high-risk mutation like *TERT* promoter mutation and *TP53* are contraindications for MIT [101]. Some investigators showed that 18.7% of PTMC are associated with advance pathologic features [102]. The fact that subset of PTMC is aggressive and the inability to detect some of the findings associated with aggressive disease preoperatively make the thyroid specialists in some countries hesitant to use these methods. Molecular testing can play a significant role in the future to choose those patients who are eligible for MIT or AS and make these treatment options more acceptable.

## 6. Conclusions

Due to advanced medical technology and easier access to health care services, the incidence of thyroid nodules has increased significantly. This in turn has led to an increase in the detection of ITNs and thyroid cancers. While many of the newly detected thyroid cancers are low-risk indolent thyroid tumors, some studies have shown that there is also a true increase in the occurrence of thyroid cancers that need further evaluation and treatment.

Most of the criteria that are required to differentiate benign from malignant tumors in ITNs or aggressive thyroid cancers cannot be detected preoperatively to guide optimal management. ATA 2015 guidelines suggest that molecular testing may be used to evaluate malignancy risk, and many practices in North America including ours have incorporated molecular testing as part of the work up for ITNs over the last decade. Our group experience at McGill university teaching hospitals has demonstrated that molecular testing can reclassify more than half of the patients with ITNs as benign and spare these patients from unnecessary diagnostic surgery. Furthermore, it can help optimize the initial management in thyroid cancers with no evidence of high-risk disease preoperatively. If thyroid FNA molecular testing is to be used for clinical purposes, results must be integrated with each nodule’s sonographic characteristics, cytologic features, patient own characteristics and risk factors for thyroid cancer, and patients’ treatment preferences.

It should be noted that these molecular tests are still not widely available and there is still a considerable amount of variability in thyroid cancer practices not only between continents, but also among different areas in the same country and among national institutions. It remains a challenge to define a universally acceptable approach for molecular testing of ITNs. Furthermore, molecular testing has seldom been studied in regions outside of North America. It is also important to highlight that no single molecular test is considered to be the gold standard and that each comes with several advantages and disadvantages. The choice of molecular test for individual practices also depends in part on regional and global differences in management paradigms, as have been historically reported between different countries. For practices with a relatively low threshold to pursue diagnostic surgery for ITNs, large multigene test panels with high NPVs would be valuable to identify those nodules that can be spared unnecessary diagnostic surgery. In contrast, for practices where clinical guidelines favor AS, a smaller panel of markers with high PPV for cancer (*BRAF V600E* single-gene test or seven-gene panel) may suffice for selecting nodules that warrant resection. While routine molecular testing is not firmly established for thyroid FNA specimens that are suspicious or positive for malignancy (Bethesda V and VI), knowledge of a thyroid nodule’s molecular risk group profile in such cases, together with its clinical and radiologic features, may help us to select the optimal surgical options (lobectomy versus upfront total thyroidectomy and central neck dissection), as demonstrated by our studies.

## Figures and Tables

**Table 1:** Potential advantages of molecular tests for thyroid nodules and advanced thyroid cancers [53,55,56,57,58].

Refining cancer probability in nodules classified as Bethesda III or IV, and guiding the decision between sonographic surveillance and diagnostic/therapeutic thyroidectomy
Helping to identify a subset of high-risk cancers, including PDTC and ATC, preoperatively
Helping to predict the cancer type and risk of cancer recurrence
Identifying potential therapeutic targets for advanced thyroid cancer (e.g., NTRK, RET, ALK, BRAFV600E)
May facilitate more individualized management of patients with Bethesda V/VI FNA cytology by guiding the extent of surgery and/or identifying possible non-surgical therapies
Screening for germline mutation associated with hereditary cancer syndrome (e.g., PTEN, RET, APC, DICER1)

## Supplementary Materials

The following supporting information can be downloaded at: https://www.mdpi.com/article/10.3390/cancers14174140/s1. File S1: Examples of Patient Report for Two of The Commercially Available Molecular Testing. Permission was granted by Dr Yuri Nikivorov to use the ThyroSeq report sample in our article. The license to use the Afirma report sample is in the following link: https://creativecommons.org/licenses/by-nc/4.0/ (accessed on 25 July 2022).
