# Supplementary figures and images for: Molecular Testing for Thyroid Nodules: The Experience at McGill University Teaching Hospitals in Canada
Source: Cancers (Basel). 2022 Aug 26;14(17):4140. doi: 10.3390/cancers14174140 (PMC9454567; doi:10.3390/cancers14174140)

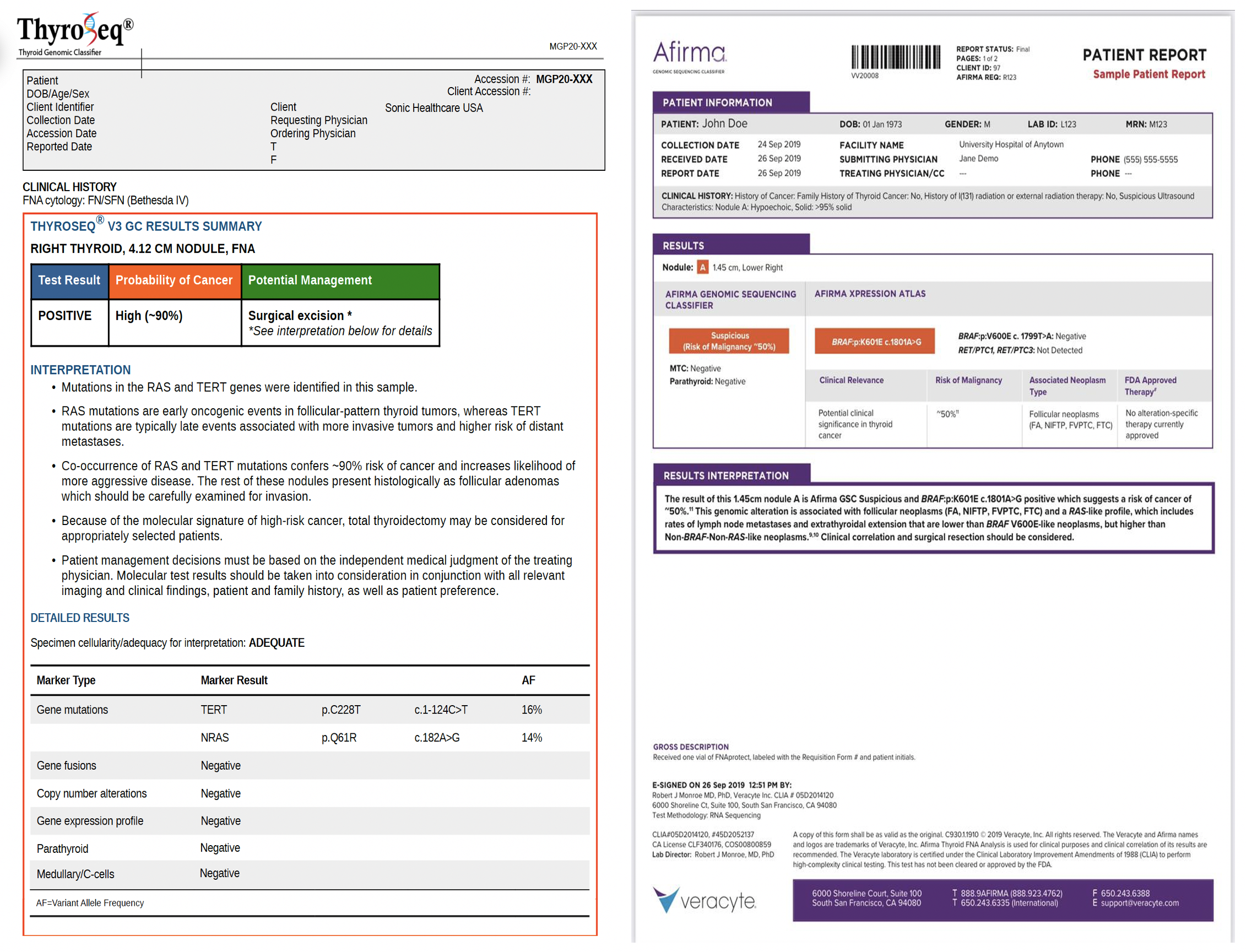

Supplement: Supplementary file 1 [file cancers-14-04140-s001.zip › Supplementary File S1/cancers-1860520-supplementary.png]
